# Supplementary material for: Risk reduction in SARS-CoV-2 infection and reinfection conferred by humoral antibody levels among essential workers during Omicron predominance
Source: PLoS One. 2024 Dec 31;19(12):e0306953. doi: 10.1371/journal.pone.0306953 (PMC11687913; doi:10.1371/journal.pone.0306953)
Supplement: S1 Table — aAll vaccine doses are monovalent origin strain WA-1 mRNA vaccines. bOther essential workers include occupation sectors with potentially high exposures to SARS-CoV-2 such as education, agriculture, public transportation services, waste collection, delivery, utilities, community-based services, childcare, and others. cChronic conditions include asthma, chronic lung disease, cancer, diabetes, heart disease, hypertension, immunosuppression, kidney disease, liver disease, neurologic or neuromuscular disease or disorder, and autoimmune disease. dParticipants are asked “Are you currently taking prednisone or other ongoing steroid medications (excluding inhaled steroids and one-time injections) or any other medications that may suppress your body’s ability to fight infection?”. eICE (immune conferring event) is the third vaccine dose for the first-time post-vaccination infection with Omicron cohort and the 3 dose strata within the reinfection cohort, the second vaccine dose for the 2 dose strata within the reinfection cohort, and the initial SARS-CoV-2 infection for the unvaccinated strata within the reinfection cohort. fExposure to individuals infected with SARS-CoV-2. gReported personal protective equipment (PPE) adherence at work was defined as the percentage of time in which an individual uses the PPE recommended by their employer when in direct contact with people. (DOCX) [file pone.0306953.s001.docx]

**S1 Table.**

| **Variable** | **Unvaccinated (n=314)** | | **2 doses^a^ (n=190)** | | **3 doses^a^ (n=196)** | |
| --- | --- | --- | --- | --- | --- | --- |
|  | Cases | Controls | Cases | Controls | Cases | Controls |
| **Site, n (%)** |  |  |  |  |  |  |
| Tucson, AZ | 55 (35.0) | 55 (35.0) | 36 (37.9) | 36 (37.9) | 32 (32.7) | 32 (32.7) |
| Phoenix, AZ | 18 (11.5) | 18 (11.5) | 18 (18.9) | 18 (18.9) | 10 (10.2) | 10 (10.2) |
| Other, AZ | 14 (8.9) | 14 (8.9) | 7 (7.4) | 7 (7.4) | 7 (7.1) | 7 (7.1) |
| Florida | 31 (19.7) | 31 (19.7) | 13 (13.7) | 13 (13.7) | 6 (6.1) | 6 (6.1) |
| Minnesota | 4 (2.5) | 4 (2.5) | 5 (5.3) | 5 (5.3) | 21 (21.4) | 21 (21.4) |
| Oregon | 4 (2.5) | 4 (2.5) | 1 (1.1) | 1 (1.1) | 0 (0.0) | 0 (0.0) |
| Texas | 7 (4.5) | 7 (4.5) | 7 (7.4) | 7 (7.4) | 8 (8.2) | 8 (8.2) |
| Utah | 24 (15.3) | 24 (15.3) | 8 (8.4) | 8 (8.4) | 14 (14.3) | 14 (14.3) |
| **Age (yrs.), mean (SD)** | 44.4 (11.4) | 43.3 (11.4) | 48.1 (10.7) | 44.5 (11.8) | 44.9 (10.7) | 45.5 (11.5) |
| **Gender, n (%)** |  |  |  |  |  |  |
| Female | 80 (51.0) | 81 (51.6) | 47 (49.5) | 62 (65.3) | 62 (63.3) | 65 (66.3) |
| Male | 76 (48.4) | 76 (48.4) | 47 (49.5) | 33 (34.7) | 36 (36.7) | 33 (33.7) |
| Other | 0 (0.0) | 0 (0.0) | 0 (0.0) | 0 (0.0) | 0 (0.0) | 0 (0.0) |
| Missing | 1 (0.6) | 0 (0.0) | 1 (1.1) | 0 (0.0) | 0 (0.0) | 0 (0.0) |
| **Race/ethnicity, n (%)** |  |  |  |  |  |  |
| Non-Hispanic, White | 98 (62.4) | 103 (65.6) | 59 (62.1) | 62 (65.3) | 75 (76.5) | 80 (81.6) |
| Hispanic | 48 (30.6) | 36 (22.9) | 26 (27.4) | 21 (22.1) | 15 (15.3) | 15 (15.3) |
| Non-Hispanic, Black | 5 (3.2) | 4 (2.5) | 3 (3.2) | 5 (5.3) | 3 (3.1) | 1 (1.0) |
| Non-Hispanic, Asian | 0 (0.0) | 3 (1.9) | 3 (3.2) | 3 (3.2) | 0 (0.0) | 1 (1.0) |
| Other | 2 (1.3) | 3 (1.9) | 0 (0.0) | 1 (1.1) | 2 (2.0) | 1 (1.0) |
| Missing | 4 (2.5) | 8 (5.1) | 4 (4.2) | 3 (3.2) | 3 (3.1) | 0 (0.0) |
| **Occupation, n (%)^b^** |  |  |  |  |  |  |
| Healthcare Worker | 38 (24.2) | 42 (26.8) | 33 (34.7) | 46 (48.4) | 54 (55.1) | 57 (58.2) |
| First Responder | 65 (41.4) | 54 (34.4) | 35 (36.8) | 27 (28.4) | 17 (17.3) | 11 (11.2) |
| Other Essential Worker | 54 (34.4) | 61 (38.9) | 27 (28.4) | 22 (23.2) | 27 (27.6) | 30 (30.6) |
| **Chronic conditions, n (%)^c^** |  |  |  |  |  |  |
| None | 111 (70.7) | 109 (69.4) | 60 (63.2) | 57 (60.0) | 66 (67.3) | 50 (51.0) |
| One | 29 (18.5) | 28 (17.8) | 23 (24.2) | 22 (23.2) | 21 (21.4) | 24 (24.5) |
| Two Or More | 11 (7.0) | 11 (7.0) | 11 (11.6) | 10 (10.5) | 11 (11.2) | 23 (23.5) |
| Missing | 6 (3.8) | 9 (5.7) | 1 (1.1) | 6 (6.3) | 0 (0.0) | 1 (1.0) |
| **Immunosuppressive medication, n (%)^d^** |  |  |  |  |  |  |
| Yes | 4 (2.5) | 5 (3.2) | 4 (4.2) | 2 (2.1) | 3 (3.1) | 3 (3.1) |
| No | 145 (92.4) | 140 (89.2) | 90 (94.7) | 86 (90.5) | 95 (96.9) | 94 (95.9) |
| Missing | 8 (5.1) | 12 (7.6) | 1 (1.1) | 7 (7.4) | 0 (0.0) | 1 (1.0) |
| **Days from ICE to blood draw, mean (SD)^e^** | 154.7 (122.7) | 182.9 (161.0) | 102.2 (98.3) | 112.7 (111.8) | 57.3 (53.6) | 59.5 (54.6) |
| **Days from ICE to infection, mean (SD)^e^** | 396.1 (198.0) | NA | 388.4 (131.8) | NA | 206.9 (108.9) | NA |
| **Avg weekly hrs. exposed to COVID, mean (SD)^f^** | 9.0 (14.0) | 6.9 (11.7) | 5.1 (10.1) | 5.7 (8.9) | 5.5 (9.2) | 5.9 (9.8) |
| **Avg % adherence to PPE rules at work, mean (SD)^g^** | 43.0 (32.4) | 45.7 (34.0) | 64.6 (24.5) | 71.0 (24.9) | 75.9 (24.8) | 78.1 (22.9) |
| **Avg % time masked in public while not at work, mean (SD)** | 26.6 (27.0) | 29.0 (31.1) | 49.2 (23.8) | 57.8 (28.0) | 58.3 (23.0) | 64.0 (24.5) |
| **Variant of first infection** |  |  |  |  |  |  |
| Origin strain WA-1 | 74 (47.1) | 74 (47.1) | 95 (100.0) | 95 (100.0) | 98 (100.0) | 98 (100.0) |
| Delta | 48 (30.6) | 48 (30.6) | 0 (0.0) | 0 (0.0) | 0 (0.0) | 0 (0.0) |
| Omicron | 35 (22.3) | 35 (22.3) | 0 (0.0) | 0 (0.0) | 0 (0.0) | 0 (0.0) |
