# Supplementary material for: Risk reduction in SARS-CoV-2 infection and reinfection conferred by humoral antibody levels among essential workers during Omicron predominance
Source: PLoS One. 2024 Dec 31;19(12):e0306953. doi: 10.1371/journal.pone.0306953 (PMC11687913; doi:10.1371/journal.pone.0306953)
Supplement: S2 Table — (DOCX) [file pone.0306953.s002.docx]

**S2 Table.**

|  | **Unvaccinated (n=314)** | | **2 doses (n=190)** | | **3 doses (n=196)** | |
| --- | --- | --- | --- | --- | --- | --- |
| **Variable** | *Cases* | *Controls* | *Cases* | *Controls* | *Cases* | *Controls* |
| **RBD End Titer, n (%)** |  |  |  |  |  |  |
| 1:60 | 35 (22.3) | 19 (12.1) | 1 (1.1) | 0 (0.0) | 0 (0.0) | 0 (0.0) |
| 1:180 | 31 (19.7) | 25 (15.9) | 0 (0.0) | 0 (0.0) | 0 (0.0) | 0 (0.0) |
| 1:540 | 39 (24.8) | 35 (22.3) | 4 (4.2) | 2 (2.1) | 0 (0.0) | 1 (1.0) |
| 1:1620 | 28 (17.8) | 34 (21.7) | 14 (14.7) | 14 (14.7) | 13 (13.3) | 3 (3.1) |
| 1:4860 | 9 (5.7) | 30 (19.1) | 15 (15.8) | 14 (14.7) | 42 (42.9) | 33 (33.7) |
| 1:9720 | 15 (9.6) | 14 (8.9) | 61 (64.2) | 65 (68.4) | 43 (43.9) | 61 (62.2) |
| **RBD AUC, mean (SD)** | 0.0047 (0.004) | 0.0060 (0.004) | 0.0128 (0.005) | 0.0132 (0.004) | 0.0114 (0.003) | 0.0125 (0.0027) |
| **S2 End Titer, n (%)** |  |  |  |  |  |  |
| 1:60 | 6 (3.8) | 2 (1.3) | 2 (2.1) | 0 (0.0) | 1 (1.0) | 0 (0.0) |
| 1:180 | 2 (1.3) | 3 (1.9) | 0 (0.0) | 1 (1.1) | 1 (1.0) | 0 (0.0) |
| 1:540 | 25 (15.9) | 22 (14.0) | 1 (1.1) | 3 (3.2) | 0 (0.0) | 0 (0.0) |
| 1:1620 | 65 (41.4) | 49 (31.2) | 14 (14.7) | 9 (9.5) | 16 (16.3) | 6 (6.1) |
| 1:4860 | 34 (21.7) | 60 (38.2) | 20 (21.1) | 25 (26.3) | 41 (41.8) | 39 (39.8) |
| 1:9720 | 25 (15.9) | 21 (13.4) | 58 (61.1) | 57 (60.0) | 39 (39.8) | 53 (54.1) |
| **S2 AUC, mean (SD)** | 0.0085 (0.003) | 0.0093 (0.003) | 0.0124 (0.004) | 0.0128 (0.003) | 0.0108 (0.002) | 0.0118 (0.002) |
