# Supplementary material for: Risk reduction in SARS-CoV-2 infection and reinfection conferred by humoral antibody levels among essential workers during Omicron predominance
Source: PLoS One. 2024 Dec 31;19(12):e0306953. doi: 10.1371/journal.pone.0306953 (PMC11687913; doi:10.1371/journal.pone.0306953)
Supplement: S3 Table — Abbreviations: OR: odds ratio; CI: confidence interval. Odds ratio represents odds of being a case for each standard deviation increase in AUC. Cases were defined as individuals who became infected with Omicron after receiving three origin strain WA-1 monovalent COVID-19 vaccine doses and no prior infections. Cases and controls were matched on number of days between blood draw and third vaccine dose, and study site. *Statistically significant at alpha = 0.05. (DOCX) [file pone.0306953.s003.docx]

**S3 Table.**

|  | **RBD AUC** | | **S2 AUC** | |
| --- | --- | --- | --- | --- |
|  | *Unadjusted OR (95% CI)* | *Adjusted OR (95% CI)* | *Unadjusted OR (95% CI)* | *Adjusted OR (95% CI)* |
| AUC | 0.78 (0.69, 0.88) | 0.77 (0.67, 0.88) | 0.80 (0.70, 0.90) | 0.79 (0.69, 0.90) |
| Age (50+) |  | 0.41 (0.31, 0.54) |  | 0.41 (0.31, 0.54) |
| Female |  | 1.12 (0.87, 1.43) |  | 1.14 (0.89, 1.46) |
| Chronic Condition |  | 1.08 (0.84, 1.41) |  | 1.11 (0.86, 1.44) |
| Hrs. Covid Exposure |  | 1.05 (0.80, 1.40) |  | 1.07 (0.80, 1.41) |
| % PPE Adherence At Work |  | 0.66 (0.50, 0.88) |  | 0.66 (0.50, 0.87) |
| % Masked In Community |  | 0.84 (0.64, 1.09) |  | 0.84 (0.64, 1.10) |
