# Supplementary material for: Risk reduction in SARS-CoV-2 infection and reinfection conferred by humoral antibody levels among essential workers during Omicron predominance
Source: PLoS One. 2024 Dec 31;19(12):e0306953. doi: 10.1371/journal.pone.0306953 (PMC11687913; doi:10.1371/journal.pone.0306953)
Supplement: S4 Table — Abbreviations: OR: odds ratio; CI: confidence interval. Odds ratio represents odds of being a case for each standard deviation increase in AUC. aCases were defined as individuals who became reinfected with SARS-CoV-2 while unvaccinated. Both cases and controls were unvaccinated at the time of their blood draw. bCases were defined as individuals who became reinfected with SARS-CoV-2. Both cases and controls were unvaccinated at time of initial infection, then received 2 doses of an origin strain WA-1 monovalent COVID-19 vaccine prior to any potential reinfection. Blood draw for both cases and controls occurred after the 2nd dose. cCases were defined as individuals who became reinfected with SARS-CoV-2. Both cases and controls were unvaccinated at time of initial infection, then received 3 doses of an origin strain WA-1 monovalent COVID-19 vaccine prior to any potential reinfection. Blood draw for both cases and controls occurred after the 3rd dose. dAt least one chronic condition versus no chronic conditions. eAbove cohort mean versus below cohort mean. *Statistically significant at alpha = 0.05. (DOCX) [file pone.0306953.s004.docx]

**S4 Table.**

|  | **RBD AUC** | | **S2 AUC** | |
| --- | --- | --- | --- | --- |
|  | *Unadjusted OR (95% CI)* | *Adjusted OR (95% CI)* | *Unadjusted OR (95% CI)* | *Adjusted OR (95% CI)* |
| **Unvaccinated (n=314)^a^** |  |  |  |  |
| AUC | 0.63 (0.46, 0.88)* | 0.64 (0.44, 0.93)* | 0.75 (0.59, 0.96)* | 0.72 (0.54, 0.94)* |
| Age 50+ |  | 1.39 (0.73, 2.65) |  | 1.34 (0.71, 2.54) |
| Female |  | 1.04 (0.58, 1.87) |  | 1.05 (0.58, 1.89) |
| Chronic Conditions^d^ |  | 0.76 (0.42, 1.38) |  | 0.78 (0.44, 1.41) |
| Hrs. COVID Exposure^e^ |  | 1.29 (0.74, 2.24) |  | 1.25 (0.72, 2.17) |
| PPE% Community^e^ |  | 0.90 (0.41, 1.96) |  | 0.89 (0.41, 1.93) |
| PPE% Work^e^ |  | 0.88 (0.45, 1.73) |  | 0.91 (0.47, 1.77) |
| **2 Doses (n=190)^b^** |  |  |  |  |
| AUC | 0.86 (0.56, 1.32) | 0.91 (0.55, 1.50) | 0.83 (0.58, 1.19) | 0.91 (0.61, 1.34) |
| Age 50+ |  | 1.54 (0.80, 2.98) |  | 1.54 (0.80, 2.97) |
| Female |  | 0.89 (0.46, 1.74) |  | 0.89 (0.46, 1.74) |
| Chronic Conditions^d^ |  | 1.01 (0.48, 2.12) |  | 1.00 (0.48, 2.10) |
| Hrs. COVID Exposure^e^ |  | 0.73 (0.36, 1.48) |  | 0.72 (0.36, 1.47) |
| PPE% Community^e^ |  | 0.97 (0.44, 2.10) |  | 0.97 (0.45, 2.09) |
| PPE% Work^e^ |  | 0.65 (0.31, 1.34) |  | 0.65 (0.31, 1.34) |
| **3 Doses (n=196)^c^** |  |  |  |  |
| AUC | 0.45 (0.25, 0.81)* | 0.51 (0.28, 0.94)* | 0.51 (0.32, 0.83)* | 0.56 (0.34, 0.92)* |
| Age 50+ |  | 1.04 (0.55, 1.98) |  | 1.04 (0.55, 1.97) |
| Female |  | 0.89 (0.46, 1.70) |  | 0.98 (0.51, 1.88) |
| Chronic Conditions^d^ |  | 0.56 (0.29, 1.09) |  | 0.55 (0.28, 1.05) |
| Hrs. COVID Exposure^e^ |  | 0.91 (0.42, 1.99) |  | 0.96 (0.44, 2.11) |
| PPE% Community^e^ |  | 0.61 (0.25, 1.47) |  | 0.56 (0.23, 1.37) |
| PPE% Work^e^ |  | 1.19 (0.48, 2.92) |  | 1.19 (0.48, 2.91) |
