# Supplementary material for: Risk reduction in SARS-CoV-2 infection and reinfection conferred by humoral antibody levels among essential workers during Omicron predominance
Source: PLoS One. 2024 Dec 31;19(12):e0306953. doi: 10.1371/journal.pone.0306953 (PMC11687913; doi:10.1371/journal.pone.0306953)
Supplement: S5 Table — Abbreviations: OR: odds ratio; CI: confidence interval. Odds ratio represents odds of being a case for each 3-fold increase in end titer. Cases were defined as individuals who became infected with Omicron after receiving three COVID-19 vaccine doses. Cases and controls were matched on number of days between blood draw and third vaccine dose, and study site. aAt least one chronic condition versus no chronic conditions. bAbove cohort mean versus below cohort mean.*Statistically significant at alpha = 0.05. (DOCX) [file pone.0306953.s005.docx]

**S5 Table.**

| Variable | **RBD End Titer** | | **S2 End Titer** | |
| --- | --- | --- | --- | --- |
|  | *Unadjusted OR*  *(95% CI)* | *Adjusted OR*  *(95% CI)* | *Unadjusted OR*  *(95% CI)* | *Adjusted OR (95% CI)* |
|  |  |  |  |  |
| End Titer | 0.82 (0.68, 0.98)* | 0.79 (0.66, 0.96)* | 0.74 (0.63, 0.87)* | 0.75 (0.63, 0.88)* |
| Age (50+) |  | 0.40 (0.30, 0.53)* |  | 0.41 (0.31, 0.55)* |
| Female |  | 1.10 (0.86, 1.41) |  | 1.13 (0.88, 1.44) |
| Chronic condition^a^ |  | 1.09 (0.84, 1.41) |  | 1.11 (0.86, 1.44) |
| Hrs. COVID exposure^b^ |  | 1.04 (0.79, 1.38) |  | 1.07 (0.80, 1.41) |
| % pPE at Work^b^ |  | 0.64 (0.49, 0.85)* |  | 0.66 (0.50, 0.88)* |
| % PPE in Community^b^ |  | 0.86 (0.66, 1.12) |  | 0.85 (0.65, 1.11) |
