# Supplementary figures and images for: Risk reduction in SARS-CoV-2 infection and reinfection conferred by humoral antibody levels among essential workers during Omicron predominance
Source: PLoS One. 2024 Dec 31;19(12):e0306953. doi: 10.1371/journal.pone.0306953 (PMC11687913; doi:10.1371/journal.pone.0306953)

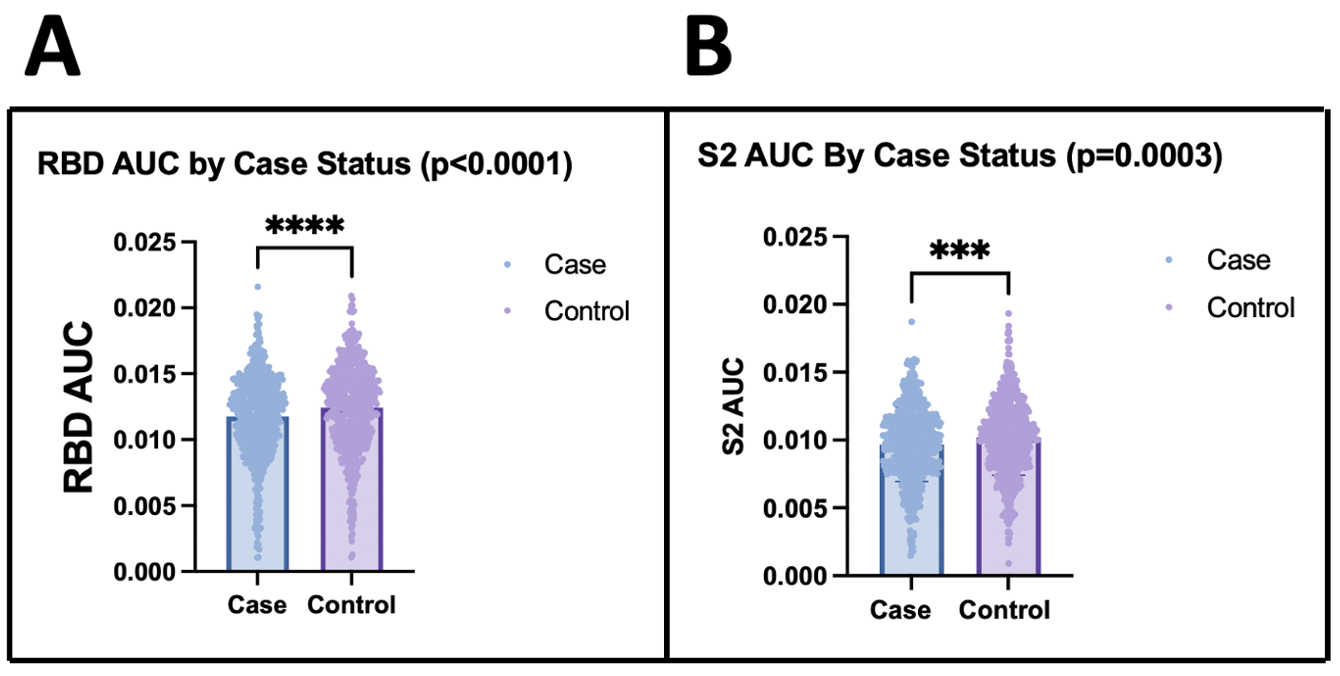

Supplement: S1 Fig — Comparison of RBD (panel A) and S2 (panel B) AUC values between cases and controls in first-time post-vaccination infection with Omicron nested case-control cohort, with difference in means evaluated by paired t-test. (TIFF) [file pone.0306953.s007.tiff]

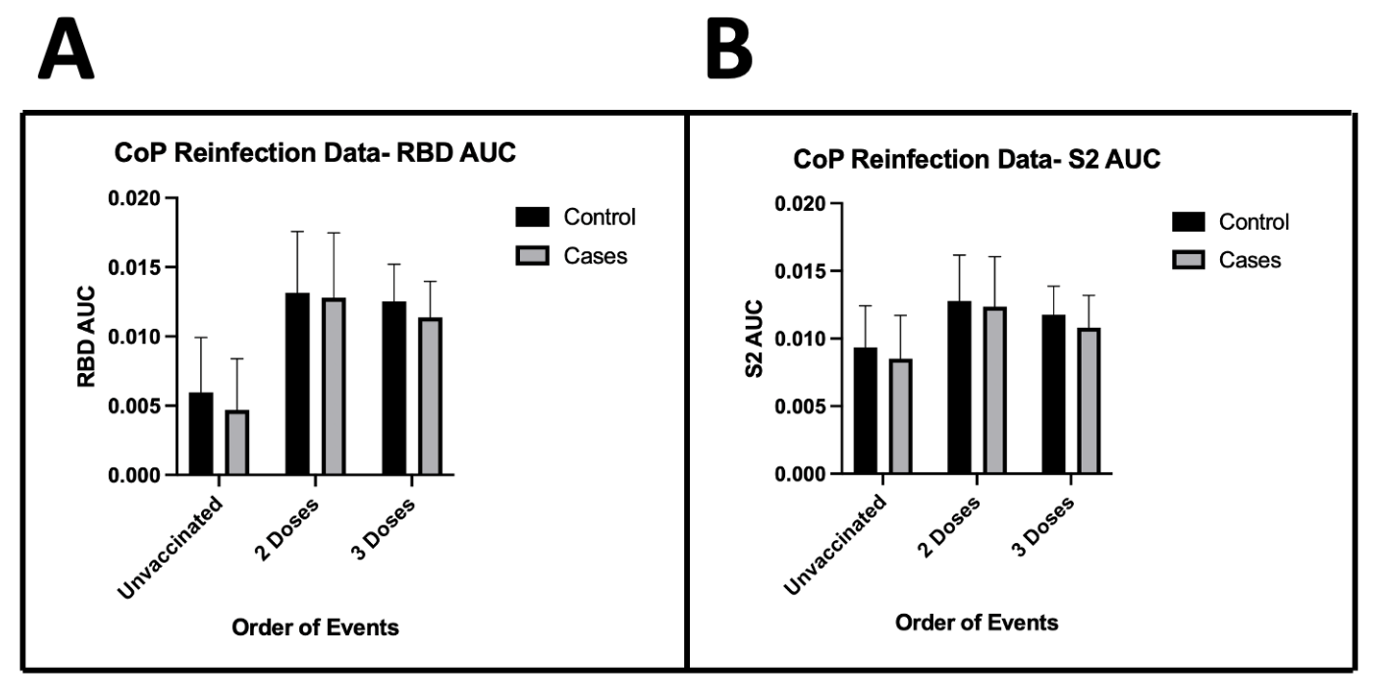

Supplement: S2 Fig — Comparison of mean RBD (panel A) and S2 (panel B) AUC values between cases and controls in reinfection Omicron infection nested case-control cohort, stratified by number of origin strain WA-1 monovalent mRNA COVID-19 vaccine doses. (TIFF) [file pone.0306953.s008.tiff]

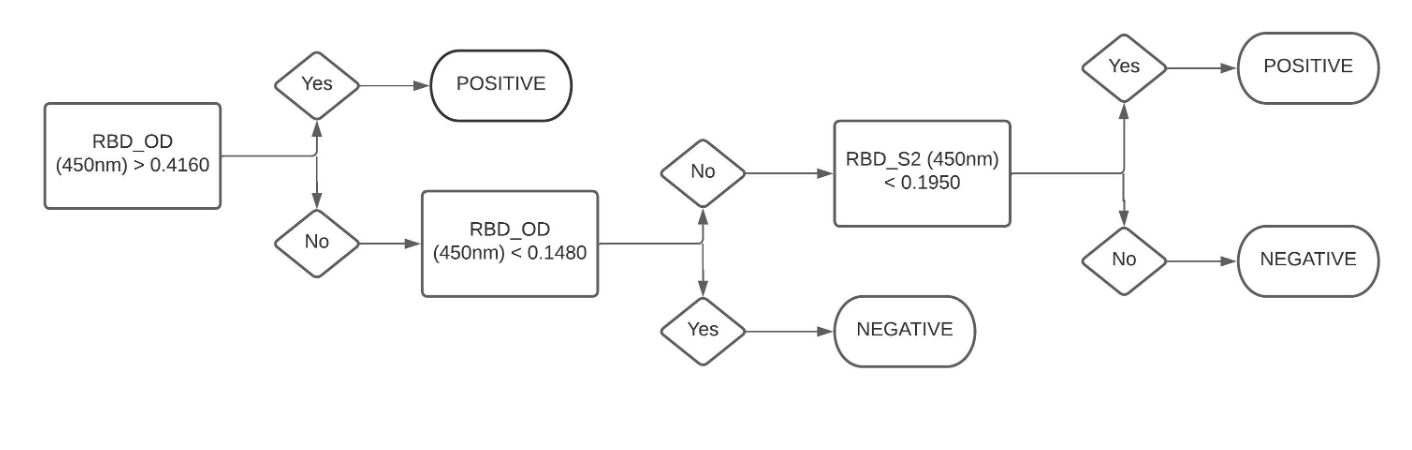

Supplement: S3 Fig — (TIFF) [file pone.0306953.s009.tiff]

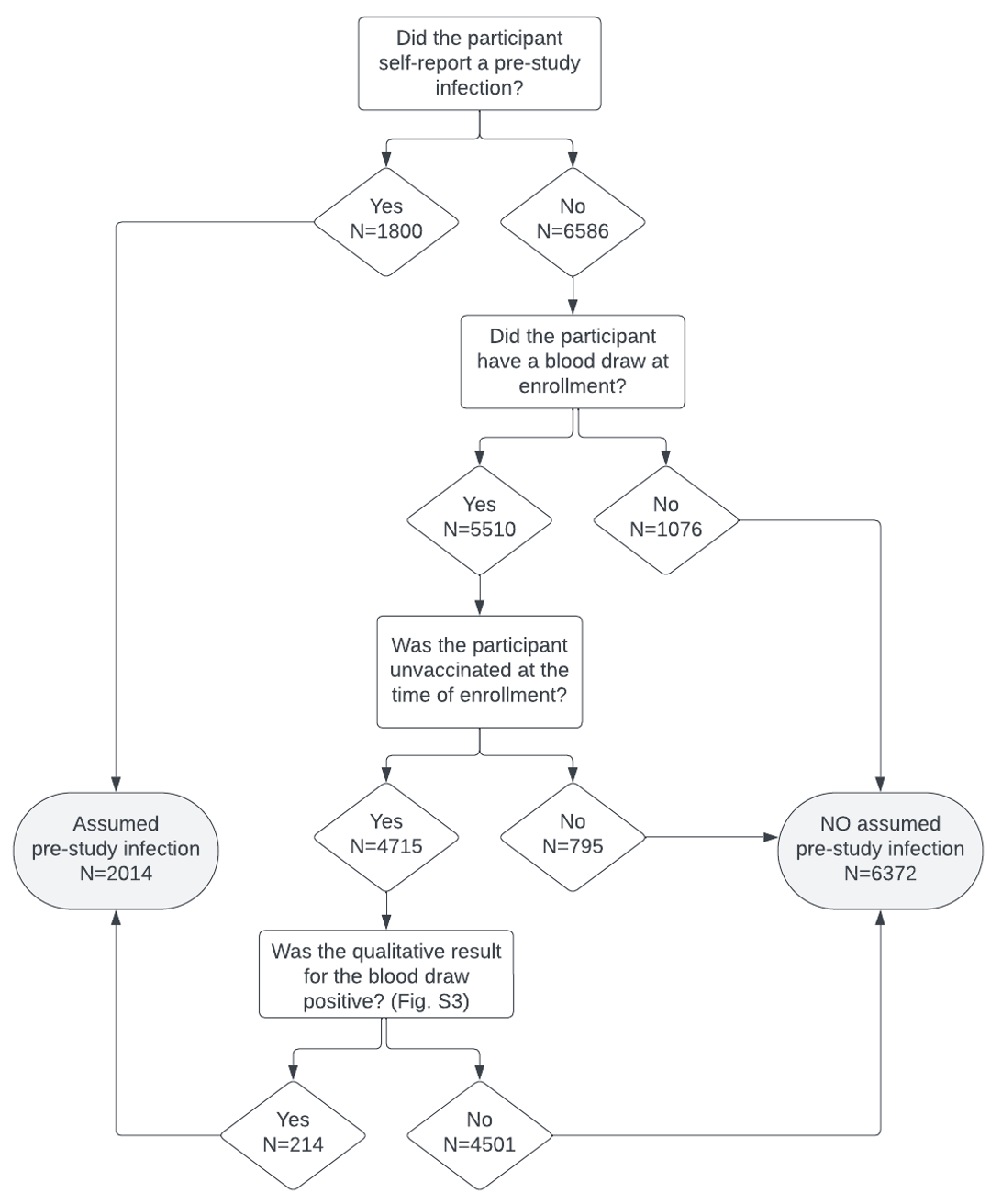

Supplement: S4 Fig — (TIFF) [file pone.0306953.s010.tiff]
