# Supplementary material for: Risk reduction in SARS-CoV-2 infection and reinfection conferred by humoral antibody levels among essential workers during Omicron predominance
Source: PLoS One. 2024 Dec 31;19(12):e0306953. doi: 10.1371/journal.pone.0306953 (PMC11687913; doi:10.1371/journal.pone.0306953)
Supplement: Attachment — Submitted filename: Response To Reviewers.docx [file pone.0306953.s011.docx]

Department of Epidemiology and Biostatistics

Roy P. Drachman Hall

1295 N. Martin Ave., Tucson, AZ 85724-5211

www.publichealth.arizona.edu

October 5, 2024

Re: Resubmission of “Risk reduction in SARS-CoV-2 infection and reinfection conferred by humoral antibody levels among essential workers during Omicron predominance”

Dear Dr. Maemu Petronella Gededzha and the PLOS ONE Editorial Staff,

Thank you for the opportunity to resubmit our manuscript “Risk reduction in SARS-CoV-2 infection and reinfection conferred by humoral antibody levels among essential workers during Omicron predominance” to PLOS ONE. In compliance with PLOS ONE publication criteria, we have made several updates to our submission. First, we have updated our Competing Interests statement to the following: “I have read the journal's policy and the authors of this manuscript have the following competing interests: RS reports a relationship with American Council of Life Insurers that includes: speaking and lecture fees. RS reports a relationship with California legal case Ebers v. Castle Park that includes: consulting or advisory. RS reports a relationship with Geneticure, Inc. that includes: equity or stocks. RS reports a relationship with Beckman Coulter that includes: speaking and lecture fees. RS reports a relationship with Shay Emma Hammer Research Foundation that includes: board membership. RS has patent issued to Arizona Board of Regents on Behalf of the University of Arizona. MG reports a relationship with Infectious Diseases and Immunization Committee, Texas Pediatric Society, Texas Chapter of the American Academy of Pediatrics that includes: board membership. This does not alter our adherence to  PLOS ONE policies on sharing data and materials.”

Second, we have updated our data sharing policies to comply with PLOS ONE publication criteria. Data cannot be shared publicly by the authors because it is owned by the Centers for Disease Control and Prevention (CDC), and the data contains personal identifying information. Data are available upon request pending approval from the CDC for researchers who meet the criteria for access to confidential data. Requests for de-identified data can be sent to LTO7@cdc.gov.

Finally, we have updated the “Methods” section of our resubmitted manuscript file. We have included our full ethics statement, and we have properly referenced all statistical software used for our analysis.

We thank the reviewers for their time in reviewing the manuscript and supplying helpful feedback. Below, we provide a point-by-point response to the reviewers’ comments and questions. In addition to this response letter, we have uploaded tracked and clean versions of the revised manuscripts.

**Reviewer #1:**

The study examined the protection conferred by antibody levels against SARS-CoV-2 in frontline workers. Higher receptor binding domain (RBD) antibody titers were linked to a reduced risk of first-time post-vaccination infection and reinfection, especially during the Omicron variant's predominance. Vaccinated individuals with higher antibody levels had a significantly lower risk of reinfection compared to unvaccinated ones. Immunity was assessed using the SARS-CoV-2 Washington-1 spike protein but the current variants and subvariants may escape immunity generated with the ancestral strain which was rightfully acknowledged as a limitation. The paper contributes to understanding the SARS-COV2 correlates of protection.

1. In line 59-60 ‘first-time post-vaccination and reinfection study samples, most were female (67%, 57%), non-Hispanic (82%, 68%), and without chronic conditions (65%, 65%)’ verify the percentages. Do the same for line 249-250.

**AUTHOR RESPONSE:** We have confirmed that these percentages are correct. These percentages were calculated by combining cases and controls to save on word count in the abstract, as well as to provide a general summary of our cohort while providing stratified information in table 1.

2. In line 308-309 ‘the 1:60 dilution, estimated risk reduction was 50% at RBD and S2 end titers between 1:540 and 1:1620 (Fig 2)’. Refer to table 2 instead of figure 2.

**AUTHOR RESPONSE**: Thank you for catching this. We actually meant to refer to figure 3 for this text, as this figure shows where the estimated risk reduction reaches 50% in this population. We have updated this in the text. In addition, we incorrectly refer to figure 3 rather than figure 4 in the reinfection results section. We have updated this in the text as well.

3. Was recurrent SARS-CoV-2 detected in any of the participants? Given the high exposure levels of the participants, one may expect reinfections to occur from the time blood was drawn.

**AUTHOR RESPONSE:** For the first-time Omicron infection cohort, we did not assess reinfections in this specific manuscript as it fell outside of the study period. However, we did observe Omicron reinfections in the general AZHEROES/RECOVER cohort, and understanding reinfections was a point of emphasis for the larger study. We invite the reviewer to review a manuscript that we previously published that assessed risk factors for Omicron reinfection among frontline workers in the AZHEROES/RECOVER cohort: <https://dx.doi.org/10.3201/eid2903.221314>.

4. Was the Omicron variant detected the same, or were various Omicron sub-variants detected in the participants? Provide details on the sequencing results.

**AUTHOR RESPONSE:** For our study set, approximately half of the nasal swabs among cases were sequenced. Among the samples that were sequenced, we identified a total of 34 unique Pango lineages for the Omicron variant, with the 5 most-common being BA.1.1, BA.1, BA.2.12.1, BA.2, and BA.5.2.1. However, because not all nasal swabs were sequenced, and because we only imputed variant of infection for those that were not sequenced (ancestral, Delta, or Omicron), we did not conduct any secondary analyses that divided our cases by lineage.

**Reviewer #2:**

The manuscript set out to report on the risk reduction and reinfection outcomes following vaccination and natural infection. The manuscript had a logical flow in information and have shown a reduction of 21% infection and 58% reinfection reduction as a result of the study vaccines used.

1. It was not clear however in the discussion as to which vaccines were used in the comparison studies that they reported on. As there some differences in vaccine type as well as manufacturer with regards to immunological response.

**AUTHOR RESPONSE**: Thank you for your review. There are differences in immunological response by vaccination manufacturer, and we agree that this is an important detail to consider when comparing results. We have added vaccine information in the second paragraph of our discussion to help contextualize the studies we are citing to compare our results.

Thank you for your consideration of this resubmission. Do not hesitate to reach out to us with questions or clarification.

Sincerely,

| **Corresponding Author**  James Hollister, MS  PhD Student, Biostatistics  University of Arizona  Mel and Enid Zuckerman College of Public Health  Email: [jameshollister@arizona.edu](mailto:jameshollister@arizona.edu) |  |
| --- | --- |
